# Typifications for *Galactia purshii* and *G. volubilis* (Fabaceae)

**DOI:** 10.3897/phytokeys.85.14935

**Published:** 2017-08-09

**Authors:** Alan R. Franck

**Affiliations:** 1 USF Herbarium, Department of Cell Biology, Microbiology, and Molecular Biology, University of South Florida, 4202 E. Fowler Avenue, ISA 2015, Tampa, FL 33620, USA

**Keywords:** *Galactia brachypoda*, *Galactia glabella*, *Galactia michauxii*, *Galactia regularis*, *Galactia volubilis*

## Abstract

The pervasive difficulties encountered with studying *Galactia* in the eastern USA necessitate additional typifications to stabilize the taxonomy. *Galactia purshii* is lectotypified here as the earliest available but overlooked name for a fairly common species of the Atlantic Coast and peninsular Florida. *Galactia volubilis* is epitypified here since the characterization of the species has been limited by its lectotype being an illustration. A lectotype is designated for Galactia pilosa var. angustifolia, a form with narrow leaves here considered synonymous with *G. volubilis*.

## Introduction

*Galactia* P.Browne (1756: 298) comprises about 100 species, of which only a few are found in the Old World (Rogers 1949, Nesom 2015). Both the genus and its species can be difficult to define. Some characters of the genus include a four-lobed calyx, papilionaceous corolla, and dehiscent fruits (Fawcett and Rendle 1920, Burkart 1971, Nesom 2015). In the USA, *Galactia* is most diverse in Texas and Florida (Nesom 2015).

The 10–15 species of *Galactia* in the eastern USA (including Florida) have had a turbulent taxonomic history, with the application of many names being excessively multifarious. Studies of type specimens and taxonomic history in *Galactia* require meticulous scrutiny. Typifications are still needed to further stabilize taxonomy in *Galactia*. A lectotype is designated for *G. purshii* Desv. and its application is discussed. An epitype is designated for *G. volubilis* (L.) Britton and a lectotype is designated for one of its synonyms, G. pilosa Nutt. var. angustifolia Torr. & A.Gray.

## Typification

### Galactia purshii

Taxon classificationPlantaeFabalesFabaceae

Desv., Ann. Sci. Nat. (Paris) 9: 413. 1826

Figs 1 , 2

Galactia purshii Desv., Ann. Sci. Nat. (Paris) 9: 413. 1826. Galactia glabella DC., Prodr. 2: 238, 1825 *nom. illeg.* (Art. 53.1) non G. glabella Michx. Fl. Bor.-Amer. (Michaux) 2: 62. 1803 *nom. illeg.* (Art. 52). **Lectotype** (designated here): Carol. [Carolina] mer. [meridionale], *Fraser s.n.* (G [G00726366]). =Galactia floridana Torr. & A.Gray var. longeracemosa Vail, Bull. Torrey Bot. Club 22: 505. 1895. **Lectotype** (designated by Nesom 2015): USA, Florida, 1889, *Simpson s.n.* (US; isolectotypes, MU, US). **syn. nov.** =Galactia michauxii A.R.Franck, Phytologia 99: 148–149. 2017. **Type**: USA, Florida, Palm Beach Co., W side of US 1, Juno Beach area, 21 Apr 1962, *Lakela 24958* (holotype, USF; isotype, FSU). **syn. nov.**

#### Remarks.

One species of *Galactia* found along the Atlantic Coast and Florida peninsula, USA, that still lacks stable nomenclature is characterized by its strigose stems that are prostrate to occasionally twining or climbing, petioles usually shorter than the terminal leaflet, leaflets often drying to a darkened or brownish color with conspicuous reticulate venation adaxially and prominent secondary venation abaxially, flower buds usually acuminate at the apex, and non-villous flowers 10–18 mm long not drying reddish that are often congested together near the apex of the inflorescence. The earliest applicable name for this species is *G. purshii*, a name chiefly ignored, and the recently introduced *G. michauxii* A.R.Franck is a synonym. This species had previously gone under several misapplied names, including *G. glabella* Michx., nom. illeg. (Pursh 1814, Nuttall 1818, Elliott 1824, Candolle 1825, Torrey and Gray 1838–1840, Chapman 1860, Britton 1881, Duncan 1979), *G. regularis* (L.) Britton et al. (Britton et al. 1888, Vail 1895, Small 1903, Small 1933, Rogers 1949, Long and Lakela 1971, Wunderlin 1982, Gleason and Cronquist 1991, Isely 1998,), *G. volubilis* (Ward and Hall 2004, Wunderlin and Hansen 2011), and *G. brachypoda* Torr. & A.Gray (Nesom 2015, 2017). Due to the scarcity of specimen citations, it is often difficult to know if the species concepts of these authors were wholly equivalent to *G. purshii* or had conflated *G. purshii* with other species.

The first description of the taxon here referred to as *G. purshii* may be attributable to Michaux (1803), who supplied a brief and somewhat insufficient description. He introduced the name *G. glabella*, probably partly based on one of his own specimens (P [P00680461], Nesom 2015: fig. 6, Franck 2017a: fig. 15), and gave its distribution as Carolina and Georgia. This sterile specimen has narrowly ovate and brownish leaflets with conspicuous reticulate venation, leafy curvaceous stems, and one leafless twining stem. The twining stem is a disconnected fragment twining around the leafy stems. Assuming all stems are of the same plant, the Michaux specimen is consistent with *G. purshii* (Duncan 1979 [as *G. glabella*], Franck 2017a [as *G. michauxii*]). The Michaux specimen is similar to other specimens of *G. purshii* that have some moderately twining stems such as *Daoud 49* (USF) from North Carolina, *Kral 11078* (USF) from Virginia, and *Seymour 91 7 20* (USF) from Virginia, each of which had been previously annotated with three different names: *G. glabella*, *G. regularis*, and *G. volubilis*. The Michaux specimen had also been identified as *G. volubilis* partly because of the twining found on the specimen (Nesom 2015). However, *G. volubilis* usually has profusely twining stems and leaflets that dry to a light green and have obscure, inconspicuous reticulate and secondary venation unlike the Michaux specimen. The leaflet shape and stem vestiture also do not seem consistent with *G. mollis* Michx. or *G. regularis*, which also occur in the Carolinas and Georgia.

Nevertheless, *G. glabella* is an illegitimate name because Michaux cited the earlier name *Ervum volubile* Walter in synonymy, thereby adopting the type of *E. volubile* (McNeill et al. 2012: Art. 52, Franck 2017a). *Ervum volubile* (=*G. glabella*) so far remains untypified. Within *Galactia* its precise application may be relatively inconsequential since its specific epithet is blocked by *G. volubilis* (L.) Britton. Additionally, the species of *Galactia* in the eastern USA are likely to remain in *Galactia* as they appear to be closely related to the type species of *Galactia*, *G. pendula* Pers., and part of a monophyletic clade based on a recent DNA phylogeny (Queiroz et al. 2015).

Pursh (1814: 487) adopted the name *G. glabella* and expanded Michaux’s description by adding the Latin terms “prostrata, subvolubilis, foliis ternatis utrinque glabris, racemis axillaribus simplicibus abbreviatis paucifloris” [prostrate, partly twining, leaflets three both sides glabrous, raceme axillary singular short few-flowered], and “leguminibus villosis” [legume villous]. With the annotation “v.v.” he noted he had made field observations of this species. Pursh stated that the flowers were “extremely pretty, purple, red and white mixed.” His description of purple flowers matches *G. purshii* but the red flower color and villous legume seem to pertain more to *G. mollis* (Radford et al. 1968: 644), possibly indicating Pursh had included more than one species in his description. Pursh gave the distribution as New Jersey to Carolina and cited *E. volubile* of Walter and *Dolichos regularis* of Willdenow (1803) in synonymy. Willdenow (1803: 1049) had simply repeated the description of *D. regularis* from Linnaeus.

Nuttall (1818: 117) continued the use of *G. glabella*, with his description closely matching that of Pursh (1814). Nuttall added that the leaves were subcoriaceous and lucid, racemes pedunculate and a little shorter than the leaves, flowers pedicellate, and legumes smooth. Under *G. mollis*, Nuttall stated that “In Herb. Muhl. [*G. mollis* was] confounded with *G. glabella*,” supporting the notion that Pursh may have also conflated *G. mollis* with his concept of *G. glabella*. *Galactia glabella* was also recognized by Elliott (1824: 239).

Candolle’s (1825: 238) Latin description of *G. glabella* was nearly verbatim of Pursh (1814), but added that the flowers were pedicellate as Nuttall (1818) had also described. Candolle mentioned that the legume was villous based on Michaux and Pursh but was glabrous based on Nuttall and his own observations. Anent this discrepancy he stated “An duae spec. confusae?” Since Michaux never described the fruits in the protologue, perhaps Candolle observed a Michaux specimen labeled *G. glabella* with villous fruits, a character which would be more like *G. mollis*. In synonymy Candolle listed *E. volubile* Walter and *D. regularis* L.

In Desvaux’s (1826) account of *Galactia*, he included descriptions for five species. For his first species he introduced the name *G. purshii*, validated solely by the description of *G. glabella* given by Candolle (1825: 238). I could find no explanation for its etymology, but it presumably honors Frederick Pursh. Desvaux considered *G. glabella* and its listed synonyms (Candolle 1825: 238) to be misapplied to the newly coined *G. purshii*. Desvaux excluded the synonyms listed by Candolle (*E. volubile* and *D. regularis*) with the abbreviation “excl. syn.” By excluding *E. volubile*, Desvaux excluded *G. glabella* since it is a superfluous name homotypic with *E. volubile*. Furthermore, for his second species, Desvaux (1826) listed and provided a separate description for *G. glabella* of Michaux, and included *G. pilosa* as its synonym. *Galactia pilosa* is currently considered a synonym of *G. mollis* and the ambiguously described *E. volubile* (=*G. glabella*) might also be conspecific with *G. mollis* (Franck 2017a). Desvaux’s description of *G. glabella* could fit the current concept of *G. mollis* or *G. volubilis*. Desvaux errantly cited page 64 instead of page 62 for the protologue of *G. glabella* Michx.

After Desvaux’s (1826) treatment, *G. purshii* was abandoned from usage, treated as a synonym, or considered illegitimate. The name *G. glabella* continued to be utilized (Torrey and Gray 1838–1840: 287, Chapman 1860: 109, Britton 1881: 27), although it is difficult to ascertain if its taxonomic concept was completely equal to the concept of *G. purshii* here. *Galactia glabella* was then considered a synonym of *G. regularis* without mention of *G. purshii* (Britton et al. 1888: 14, Jackson 1893: 987, Small 1903: 650, Small 1933: 719, Long and Lakela 1971: 493, Gleason and Cronquist 1991: 305, Isely 1998: 569). Vail (1895) listed both *G. glabella* and *G. purshii* as synonyms of *G. regularis*. Duncan (1979) resurrected the use of *G. glabella*, including *G. purshii* in synonymy and separating it from *G. regularis*, and some specimens at USF were annotated by Nesom as *G. glabella*. Ward and Hall (2004) included *G. glabella* as a synonym of *G. volubilis*.

**Figure 1.**
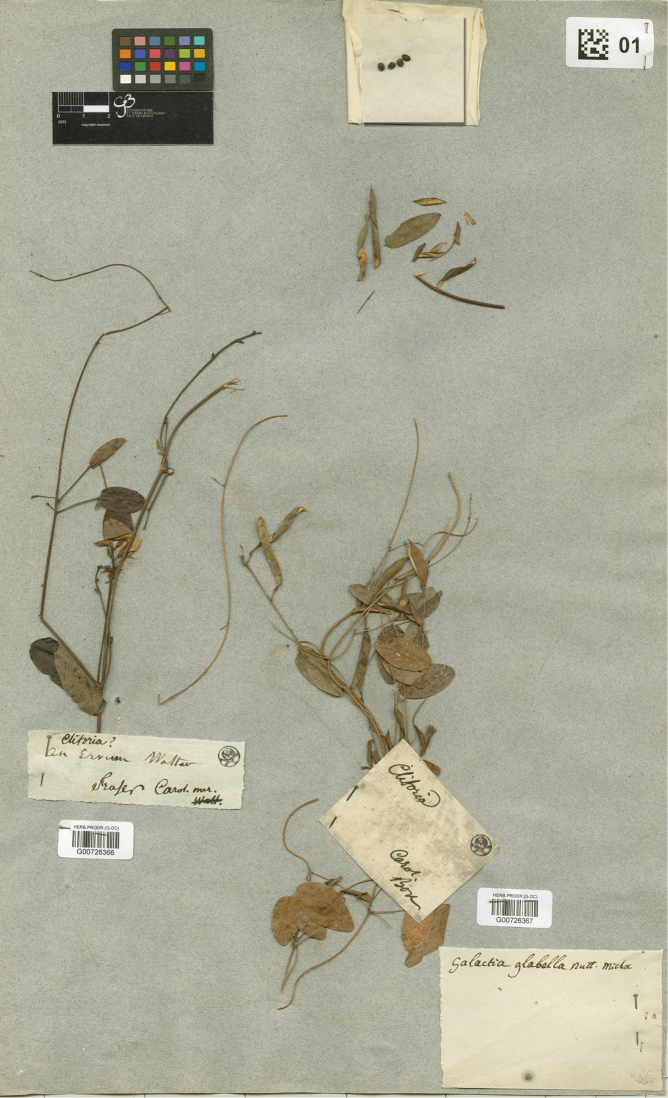
Sheet at G labeled *Galactia glabella* by Candolle. The specimen on the left is the lectotype of *G. purshii* (G00726366). The specimen on the right is *G. volubilis* (G00726367). Conservatoire et Jardin botaniques de la Ville de Genève.

*Galactia purshii* is a legitimate name since its protologue unequivocally excluded *D. regularis*, *E. volubile*, and *G. glabella*. Since *G. purshii* is validated by Candolle’s description (1825: 238), any specimens seen by Candolle for his treatment should be considered original material (McNeill et al. 2012: Arts. 7.7 and 9.3, note 3). Candolle’s (1825) annotation “(v.s.)” indicated he had seen specimens. There are two specimens in the Candolle herbarium together on one sheet, labeled in Candolle’s handwriting (Burdet 2017) as “*Galactia glabella* Nutt. Michx.” (Fig. 1). The specimen on the right (G00726367) is *G. volubilis*, twining and with ovate lightly glaucous leaflets drying pale greenish with inconspicuous secondary and reticulate venation. The John Fraser specimen on the left (G00726366, Fig. 2) is consistent with *G. glabella* sensu Candolle (1825); it has leaflets drying to a dark brown adaxially with conspicuous abaxial secondary and reticulate venation, and flowers ca. 13 mm long. The indumentum of the stem is retrorsely strigose and of the calyx abaxially antrorse but scant (L. Gautier, pers. comm.). A phrase written on the label “An *Ervum* Walter” indicated an association with the modern sense of *Galactia*. This Fraser specimen must have come from L’Héritier’s herbarium, which was purchased by Candolle in 1805 (Gray 1889, Stafleu 1966, Brummitt 1972), and can be considered part of the original material. The Fraser specimen (Figs 1–2) is designated here as the lectotype of *G. purshii*. The specimens cited for *G. michauxii* by Franck (2017a: Appendix 1) are here identified as *G. purshii*, with *G. michauxii* being a later synonym.

Recent descriptions for *G. fasciculata* Vail, such as having strigose stems (Nesom 2015, 2017), may apply to specimens here considered to be *G. purshii*. However, the indumentum of the type specimens of *G. fasciculata* appears more similar to the villous stems of *G. floridana* (Isely 1998, Franck 2017a). *Galactia fasciculata* was described as prostrate or climbing high by Vail (1895), whereas Nesom (2015, 2017) described it as high-climbing with coiling stems. The holotype label stated “climbing on small shrubs.” Additional study is needed to determine if a high-climbing habit is a reliable and distinctive character since it can only be confidently ascertained from living plants and field observations. Ward and Hall (2004) considered *G. fasciculata* very rare while Nesom (2015, 2017) considered it an endemic of central peninsular Florida.

The stem indumentum of the holotype of G. floridana Torr. & A.Gray var. longeracemosa Vail does not appear to be villous like *G. floridana*, but appears more like *G. purshii*. Galactia floridana var. longeracemosa is considered here to be a synonym of *G. purshii*. The holotype of G. floridana var. longeracemosa was probably collected by Joseph H. Simpson relatively near to Bradenton, Manatee Co., Florida where he had lived (Small 1919; Harper 1948).

The name *G. brachypoda* was apparently misapplied (Nesom 2015, 2017) to specimens here considered to be *G. purshii*. *Galactia brachypoda* was first described by Torrey and Gray (1838–1840), who indicated the habit as not twining with a two foot long flexuous stem, the calyx villous, and the flowers half as large as *G. glabella* (*G. glabella* sensu Torrey & Gray probably being misapplied to *G. purshii*). Chapman (1860), who collected the two type specimens of *G. brachypoda* (NY), described it as erect, 1–1.5 feet high and with a woolly calyx, noting his descriptions were “all my own, copying no one, when I knew the plant” (Chapman 1839–1890: 4 Apr 1959). On the label of the presumed holotype Chapman wrote “seems to come between *G. mollis* & *G. sessiliflora* [=*G. erecta* (Walt.) Vail]” which was later crossed out by a different pen, possibly by Torrey who also added what appears to be “*brevipedunculata* n. sp.” to the same label. Vail (1895) described its calyx as “clothed with spreading” hairs, the lower calyx lobes acutish, corolla 8–10 mm long, and the “vexillum” 7–8 mm long, which Small (1903) mostly repeated. All of these observations are consistent with the type specimens of *G. brachypoda*, none of which match the concept of *G. purshii*.

The habit of *G. brachypoda* has sometimes been described as similar to *G. purshii*. However, the descriptions of a decumbent (Vail 1895, Small 1903, Small 1933), ascending or sprawling (Isely 1986), procumbent (Nesom 2015), or prostrate habit (Nesom 2017) for *G. brachypoda* appear to be based on speculation from specimens and not field observations. While it was conjectured that it was impossible for *G. brachypoda* to be erect because its type specimens had stems to 37 cm long (Nesom 2017), another collection identified as *G. brachypoda* (*Anderson 15642* [FSU, GA]) with stems well over 40 cm long described on its label “robust, erect plants with limited twining” and was noted to be very similar to the type specimens of *G. brachypoda* (Franck 2017a, Nesom 2017). Furthermore, stems of some specimens of the erect *G. erecta* can reach 32–36 cm long (e.g. *Biltmore 3956a* [NY], *Horn 1032* [DUKE], *Orzell & Bridges 14271* [USF], *Rugel 150* [NY]). It does not appear to be impossible for *G. brachypoda* to be erect and have stems to 37 cm long. The characterization of *G. brachypoda* as erect by Chapman (1860) and *Anderson 15642* is considered here to be accurate.

Numerous authors noted a semblance of *G. brachypoda* with *G. erecta* (Rogers 1949, Ward and Craighead 1990, Isely 1998, Ward and Hall 2004, Franck 2017a), while others also noted a similarity to *G. mollis* (Chapman’s notes on the holotype of *G. brachypoda*, label notes of *Anderson 15642*, Franck 2017a). The acutely-tipped flower buds and relatively small reddish-drying flowers of the type specimens of *G. brachypoda* are features shared with *G. erecta* and *G. mollis*. The erect habit and elliptic leaflets with relatively long petioles of the type specimens of *G. brachypoda* are more similar to *G. erecta*. If the inflorescences of the type specimens of *G. brachypoda* are interpreted as immature (Nesom 2017), the sizes of the flower buds and corolla (including the individually mounted petals of the holotype) are still rather small compared to *G. purshii*. The long stems, pedunculate inflorescences, and villous calyces of the type specimens of *G. brachypoda* are more similar to *G. mollis*. However, inflorescences of *G. erecta* can occasionally be pedunculate, with a peduncle to 14 mm long in *Harper s.n.* (NY [02569186]). Nesom (2017) characterized the calyx of *G. brachypoda* as “very sparse,” dissimilar to other observations of the calyx as villous (Torrey and Gray 1838–1840), woolly (Chapman 1860), or “clothed with spreading” hairs (Vail 1895). Another rather odd specimen (*Duncan 17113* [GA]) seems to mix features of *G. erecta* and *G. mollis* in that it has subsessile inflorescences and long petioles like *G. erecta* and long, partly twining stems like *G. mollis*. Lastly, the left-most plant of a Chapman collection at MO (793008) appears erect like *G. erecta* but has shortly pedunculate inflorescences and indumentum more like *G. mollis*.

There are two known type specimens of *G. brachypoda* at NY (00008088 and 00008090), although there is a third specimen (NY [00008089]) that was labeled as *G. brachypoda* in Chapman’s handwriting. This third specimen is clearly *G. erecta*. It had been proposed that other authors were attempting to make two species out of *G. erecta* with the use of the name *G. brachypoda* through the study of this *G. erecta* specimen labeled as *G. brachypoda* (Nesom 2015, 2017). This specimen consists of plants ca. 13 cm tall with subsessile inflorescences. Among authors who recognized both *G. brachypoda* and *G. erecta*, this specimen matches their concepts of *G. erecta*, and is clearly incongruent with their concepts of *G. brachypoda* (Torrey and Gray 1838–1840, as *G. sessiliflora* Chapm., Chapman 1860, Vail 1895, Small 1903). The discordance of this specimen with Chapman’s (1860) concept of *G. brachypoda* suggests the possibility of a labeling error. Vail annotated the holotype of *G. brachypoda*, but not this *G. erecta* specimen. This *G. erecta* specimen was otherwise annotated only by Anita F. Cholewa in 1986, erroneously as a probable isotype of *G. brachypoda*. When Isely (1986) mentioned that *G. brachypoda* could be a “freak form” of *G. erecta*, he also stated that there were “two Chapman sheets [of *G. brachypoda*] at NY” and that *G. brachypoda* had pedunculate inflorescences, unlike this *G. erecta* specimen. Ward and Craighead (1990) speculated *G. brachypoda* was “probably an aberrant form” of *G. erecta*, and later Ward and Hall (2004) also stated that *G. brachypoda* was “based upon two A.W. Chapman specimens (NY).” The evidence does not support the idea that this specimen (NY [00008089]) nor any other of *G. erecta* was used to formulate concepts of *G. brachypoda*. I concur with previous botanists that *G. brachypoda* is closely related to *G. erecta* and *G. mollis*, and numerous features associated with the type specimens of *G. brachypoda* (i.e., its erect habit, elliptic leaflets on a long petiole, acutely-tipped flower buds, villous calyx, and relatively small reddish-drying flowers) are inconsistent with *G. purshii*.

**Fgure 2.**
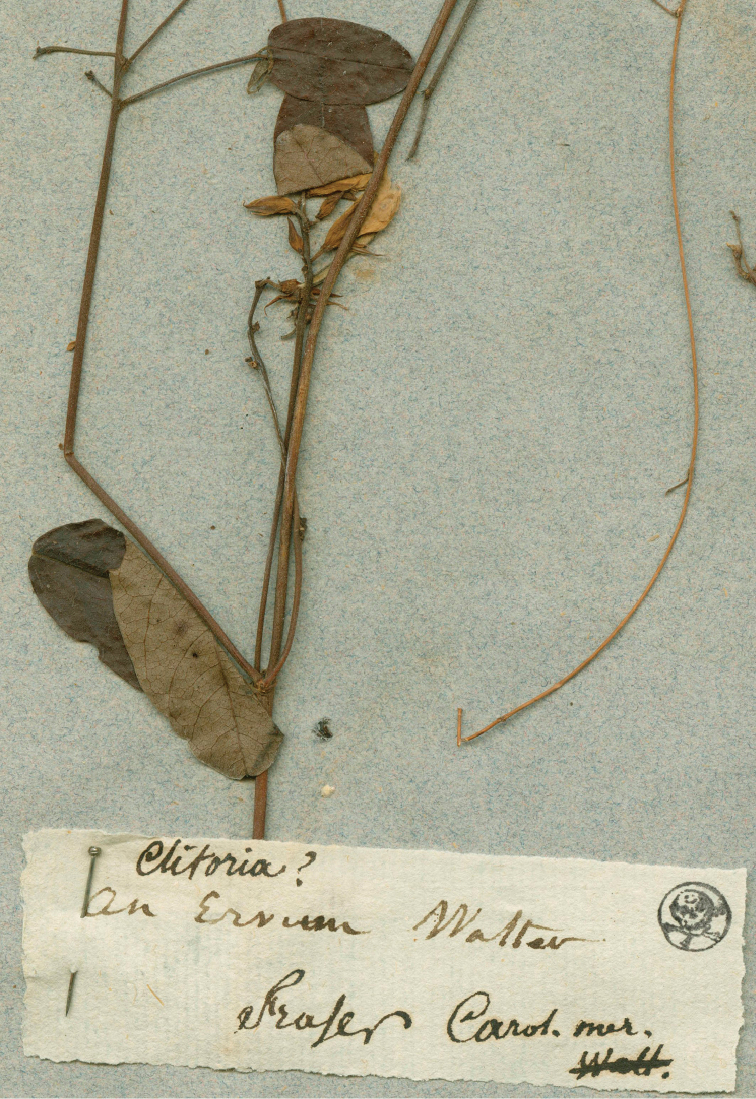
Close-up of the lectotype of *G. purshii* (G00726366). The stem fragment on the right appears to be from the specimen of *G. volubilis*. Conservatoire et Jardin botaniques de la Ville de Genève.

### Galactia volubilis

Taxon classificationPlantaeFabalesFabaceae

(L.) Britton, Mem. Torrey Bot. Club 5: 208. 1894

Figs 3 , 4

Galactia volubilis (L.) Britton, Mem. Torrey Bot. Club 5: 208. 1894. Hedysarum volubile L., Sp. Pl. 2: 750. 1753. **Lectotype** (designated by Reveal & Jarvis 2009: 979): Dillenius, Hort. Eltham. t. 143., f. 170. 1732. **Epitype** (designated here): USA, Florida, Lafayette Co., NFMYC [North Florida Methodist Youth Camp, Mayo], 13 Jun 1964, *Caudle et al. 5744* (epitype, USF; probable isoepitypes, *Caudle et al. 5292A*, FLAS, *Caudle et al. 5292B*, FTG). =Galactia macreei M.A.Curtis, Boston J. Nat. Hist. 1: 120. 1835. Galactia pilosa Nutt. var. macreei (M.A.Curtis) Torr. & A.Gray, Fl. N. Amer. 1: 287. 1838. **Type**: USA, North Carolina, *Curtis s.n.* (probable holotype, GH [00002425], photograph in Rogers 1949: 88, pl. 22). =Galactia pilosa Nutt. var. angustifolia Torr. & A.Gray, Fl. N. Amer. 1: 287. 1838. Galactia volubilis (L.) Britton var. intermedia Vail, Bull. Torrey Bot. Club 22: 508. 1895 *nom. illeg.* (Art. 52). **Lectotype** (designated here): USA, Florida, *Lt. Alden s.n.* (lectotype, NY [02569414]).

#### Remarks.

Precise measurements of flower size and plant indumentum can be essential towards the application of names in *Galactia* as demonstrated by Duncan (1979) for *G. regularis*. Since *G. volubilis* is lectotypified by an illustration, an epitype would be useful to help secure the application of *G. volubilis* and further allay any possible confusion with other taxa. No information is known for the provenance of the *G. volubilis* plant in Sherard’s garden, which was used for the Dillenius lectotype illustration. An epitype was sought here that closely matches the morphology supplied by Dillenius.

**Figure 3.**
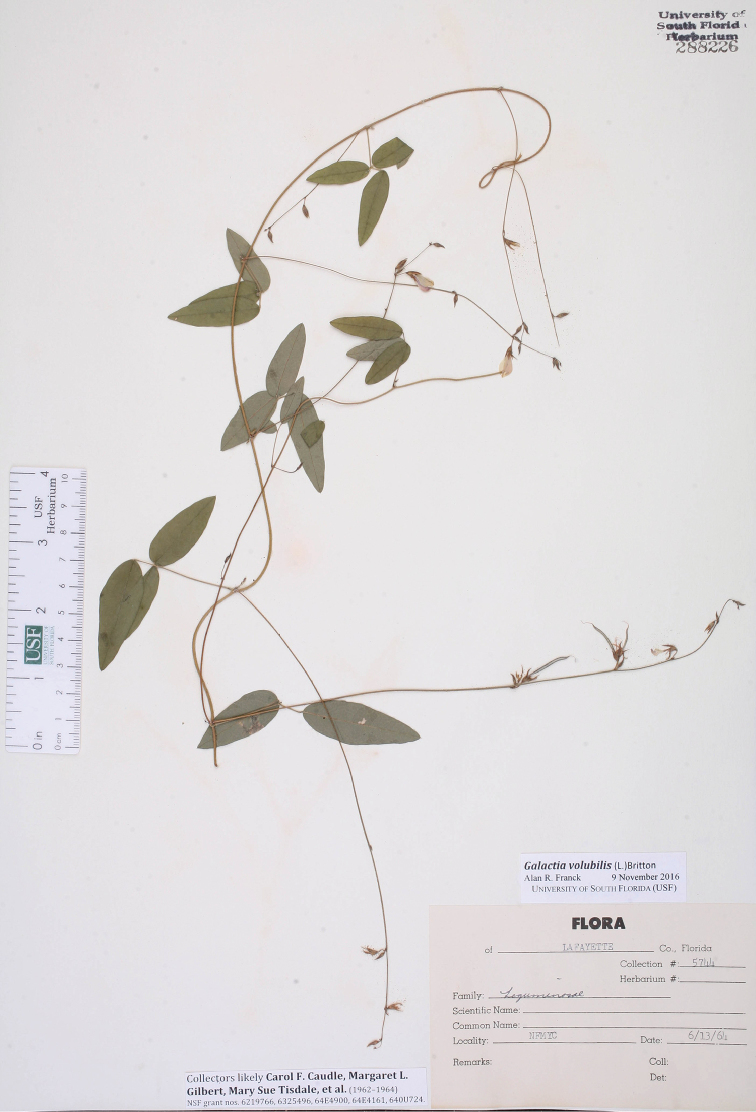
Epitype of *G. volubilis* at USF.

*Caudle et al. 5744* (USF) (Fig. 3) is chosen here as the epitype of *G. volubilis* because it is very consistent with the Dillenius lectotype and the description given by Dillenius (1732). The stems of both the lectotype and epitype are moderately sinuous with retrorsely hirsute indumentum. A close-up image of the stem indumentum of *Caudle et al. 5744* is given in Franck (2017a: fig. 40). The leaflets are lanceolate-ovate in both the lectotype and epitype. The leaflets of the epitype are glaucescent abaxially consistent with the Dillenius (1732) description “prona pallidiora & glaucescentia.” The adaxial secondary venation of the leaflets is discernible while reticulate venation is obscured in both the lectotype and epitype. The flower length is ca. 70% of the maximum leaflet width in the lectotype and ca. 75% in the epitype. The flower fascicles of both are relatively distant along the inflorescence.

Two other specimens, *Caudle et al. 5292A* and *5292B*, are very likely isoepitypes. In all features, they are markedly identical in morphology to the epitype, including the development of inflorescences with only immature fruits. The collection numbers of the labels are different but they do not appear to be traditional collection numbers. The specimens were gathered by several undergraduate students as part of a few National Science Foundation (NSF) grants awarded to Margaret L. Gilbert, the curator of the Florida Southern College herbarium (FSCL, now incorporated into USF). It appears this sequence of collection numbers was given to any specimen under the purview of these NSF grants, and were simply sequentially added as the specimens were processed back at the herbarium. It seems likely that these *G. volubilis* specimens were gathered by one group of students but later processed separately resulting in their sequential separation. Although several students and the curator were all involved with the field work, Carol F. Caudle (now Carol Baskin) related that she herself was the main person responsible for the herbarium specimens (Franck 2017b). No collectors were named on the original label but Caudle has been credited as the probable main collector.

**Figure 4.**
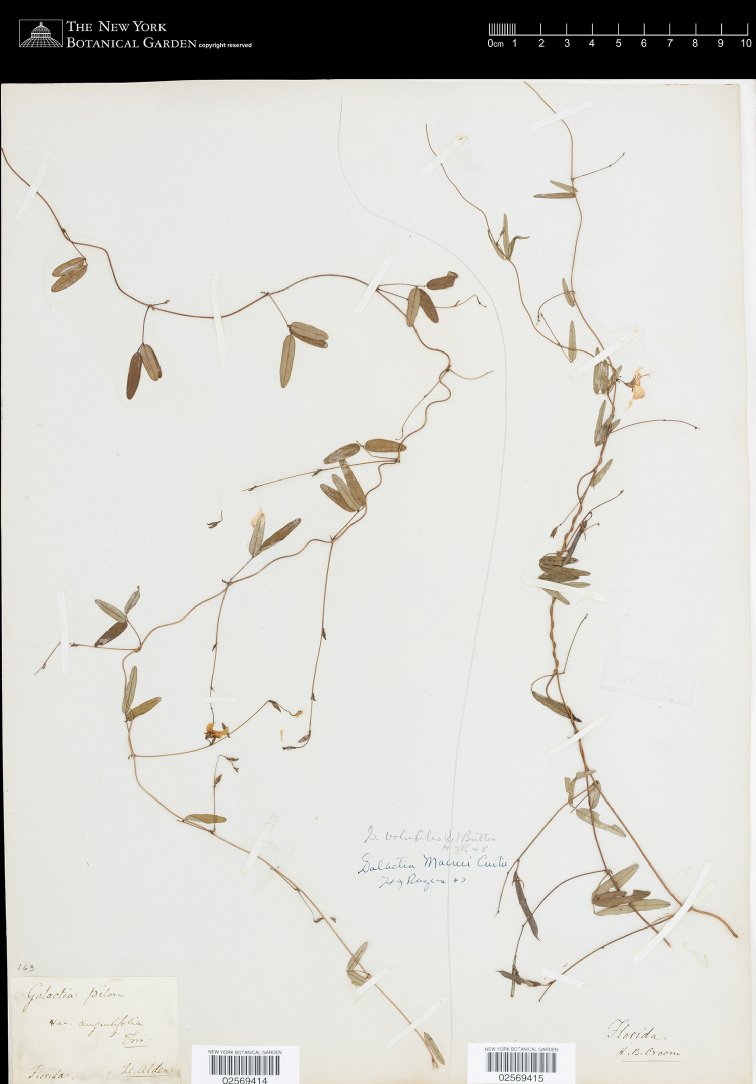
The Alden specimen on the left is selected as the lectotype of G. pilosa var. angustifolia; the specimen on the right was collected by Croom. This image belongs to The C. V. Starr Virtual Herbarium (http://sweetgum.nybg.org/science/vh/).

The concept of *G. pilosa* sensu Torrey and Gray (1838–1840) matches the sense of *G. volubilis* here (Franck 2017a). One variety introduced by Torrey and Gray was “γ. *angustifolia*” and its range was given as “δ. Middle Florida, *Croom*! East Florida, *Lieut. Alden*!” The mismatched greek symbols, γ for the name and δ for the range, must have been an error. There is a sheet at NY with both syntypes mounted on it that was annotated by Rogers as *G. macreei* in 1947 and *G. volubilis* in 1948 (Fig. 4). On the right is the Croom specimen (NY [02569415]), which likely came from near Croom’s properties in the Florida panhandle (Troyer 2002). On the left is the Alden specimen (NY [02569414]), which is labeled as Galactia pilosa var. angustifolia. Alden was stationed at Fort Brooke (Tampa, Florida) in 1832 and Fort King (Ocala, Florida) from 1832–1833 (Cullum 1891: 488; Harper 1948), and his specimen likely came from near these areas. The leaflet shapes of both specimens are narrowly oblong-ovate, being 3–6 times as long as wide and usually being widest near the base of the leaflet. The Alden specimen is selected here as the lectotype since it exemplifies the exserted long inflorescence and distantly spaced flower fascicles of *G. volubilis*.

The leaflet shape of G. pilosa var. angustifolia approaches *G. austrofloridensis* A.R.Franck, but fits within the variation of *G. volubilis*, matching other specimens with narrow leaflets such as *Correll 51775* (USF), *Hansen 5972* (USF), *Hansen 9896* (USF), and *Popenoe 2080* (USF). The linear-oblong leaflets (> 4 times as long as wide) of *G. austrofloridensis* only subtly distinguish it from *G. volubilis*. One collection from the West Indies, *Correll & Correll 47675* (FTG, NY) from the Bahamas, appears identifiable as *G. austrofloridensis*. *Galactia grisebachii* Urb., possibly endemic to Cuba (e.g. *León 7461* [NY]), has similarly linear-oblong leaflets (Nesom 2017) but seems to differ by its consistently short inflorescences (Franck 2017a). The poorly characterized *Galactia parvifolia* A.Rich, of the Greater Antilles and Bahamas, is similar to *G. grisebachii* but may differ by its lateral leaflets often being ca. ½ as long as the terminal leaflet (Urban 1900, Franck 2017a).

## Supplementary Material

XML Treatment for Galactia purshii

XML Treatment for Galactia volubilis
